# Role of uL3 in Multidrug Resistance in p53-Mutated Lung Cancer Cells

**DOI:** 10.3390/ijms18030547

**Published:** 2017-03-03

**Authors:** Annapina Russo, Assunta Saide, Silvia Smaldone, Raffaella Faraonio, Giulia Russo

**Affiliations:** 1 Department of Pharmacy, University of Naples “Federico II”, Via Domenico Montesano 49, 80131 Naples, Italy; assuntasaide@gmail.com; 2 Department of Pharmacology and Systems Therapeutics, Icahn School of Medicine at Mount Sinai, New York, NY 10029, USA; silvia.smaldone@gmail.com; 3 Department of Molecular Medicine and Medical Biotechnologies, University of Naples “Federico II”, Via Sergio Pansini 5, 80131 Naples, Italy; raffaella.faraonio@unina.it

**Keywords:** ribosomal protein, nucleolar stress, uL3, multidrug resistance, lung cancer, *Nrf2*, *xCT*, *GST-α1*, *MDR1*, chemoresistance

## Abstract

Cancer is one of the most common causes of death among adults. Chemotherapy is crucial in determining patient survival and quality of life. However, the development of multidrug resistance (MDR) continues to pose a significant challenge in the management of cancer. In this study, we analyzed the role of human ribosomal protein uL3 (formerly rpL3) in multidrug resistance. Our studies revealed that uL3 is a key determinant of multidrug resistance in *p53*-mutated lung cancer cells by controlling the cell redox status. We established and characterized a multidrug resistant Calu-6 cell line. We found that uL3 down-regulation correlates positively with multidrug resistance. Restoration of the uL3 protein level re-sensitized the resistant cells to the drug by regulating the reactive oxygen species (ROS) levels, glutathione content, glutamate release, and cystine uptake. Chromatin immunoprecipitation experiments and luciferase assays demonstrated that uL3 coordinated the expression of stress-response genes acting as transcriptional repressors of solute carrier family 7 member 11 (*xCT*) and glutathione *S*-transferase α1 (*GST-α1*), independently of Nuclear factor erythroid 2-related factor 2 (Nrf2). Altogether our results describe a new function of uL3 as a regulator of oxidative stress response genes and advance our understanding of the molecular mechanisms underlying multidrug resistance in cancers.

## 1. Introduction

Lung cancer is one of the most common causes of cancer-related death among adults [1]. Chemotherapy is recognized as an important component of treatment for all stages of lung cancer and is crucial in determining patient survival and quality of life [2]. However, intrinsic or acquired multidrug resistance (MDR) is the main reason for tumor recurrence [3]. Therefore, it is crucial to understand the molecular basis underlying the mechanisms of MDR. Here, we used 5-Fluorouracil (5-FU) to establish a multidrug resistant lung cell model [4,5].

In addition to its effect on thymidylate synthase inhibition and DNA synthesis, 5-FU may also influence RNA metabolism [6]. It is known that the nucleolus is a ribosome-supplying organelle, however a growing body of evidence suggests that the nucleolus plays intriguing novel roles in sensing cellular stress signals [7]. Under stress conditions, the structure of the nucleolus is perturbed and some nucleolar proteins, including ribosomal proteins (rp), are released from the nucleolus to the nucleoplasm, where they associate with Mouse Double Minute 2 (MDM2) to inhibit its activity and stabilize p53 [7]. In yeast, it has been reported that the nucleolus can function as an oxidative stress sensor [8].

It has been demonstrated that some rp play a key role in cell resistance to chemotherapeutic drugs [9]. Specifically, upon 5-FU induced nucleolar stress, a subset of rp including uL18 (rpL5), uL5 (rpL11), and uL14 (rpL23) are released from the ribosome to activate p53 through MDM2 inhibition [10]. Recently, our studies focused on post-transcriptional regulation [11,12,13,14] and extra-ribosomal functions of ribosomal proteins [11,15]. In particular, we demonstrated that uL3 (formerly rpL3) [16] is involved in the regulation of its own expression by regulating the splicing of its own transcript [17,18]. In addition, we identified a new molecular pathway activated in the cell response to 5-FU that is p53-independent but still rp-dependent. We demonstrated that upon ribosomal stress induced by 5-FU, ribosome free uL3 becomes a regulator of p21 [19,20], cystathionine-β-synthase (CBS) [21,22,23], and NFκB [22] expression and mediates apoptosis through the activation of the mitochondrial apoptotic response pathway. Of note, our data indicated that uL3 status is associated to chemoresistance since the loss of uL3 makes chemotherapeutic drugs, such as 5-FU, oxaliplatin (L-OHP), Actinomycin D, and Cisplatin ineffective in colon and lung cancer cells lacking active p53 [21,22,23,24]. According to this, we reported that the resistance of A549 lung cancer cells to Cisplatin correlates to the loss of uL3 expression [25]. Evidence of the role of some rp mediating drug resistance has been previously reported. To date, eL36 (rpL36) plays a role in cisplatin resistance in human carcinoma cells [26], the radiosensitivity in NSCLC (non-small cell lung cancer) cells is regulated by uS3 (rpS3) [27], and eL24 (rpL24) may have effects on drug resistance mechanisms in hepatocellular carcinoma HepG2 cells [28].

In the present study, we identified uL3 (formerly rpL3) as a key molecule conferring multidrug resistance to lung cancer cells lacking p53 and elucidated the molecular mechanism involved in this process. We have established and characterized a *p53*-mutated lung cancer cell line resistant to 5-FU. We have demonstrated that in these cells, uL3 expression is down-regulated upon acquired multidrug resistance but its restoration re-sensitized the cells to 5-FU through the regulation of reactive oxygen species (ROS) levels, glutathione (GSH) content, glutamate release, and cystine uptake. The molecular mechanism by which this occurs involves uL3-mediated control of the stress-response gene expression. Specifically, uL3 functions as a transcriptional regulator of solute carrier family 7 member 11 (*xCT*) and glutathione S-transferase α1 *(GST-α1*), independently of Nuclear factor erythroid 2-related factor 2 (Nrf2).

These data imply that there is a mechanistic link between the response pathway to drug-induced ribosomal stress and the development of drug resistance and suggest the possibility of targeting uL3 to modulate the redox status of cancer cells for future therapeutic purposes in order to overcome MDR.

## 2. Results

### 2.1. Production of the MDR Resistant p53-Mutated Lung Cancer Cell Line

**Figure 1:**
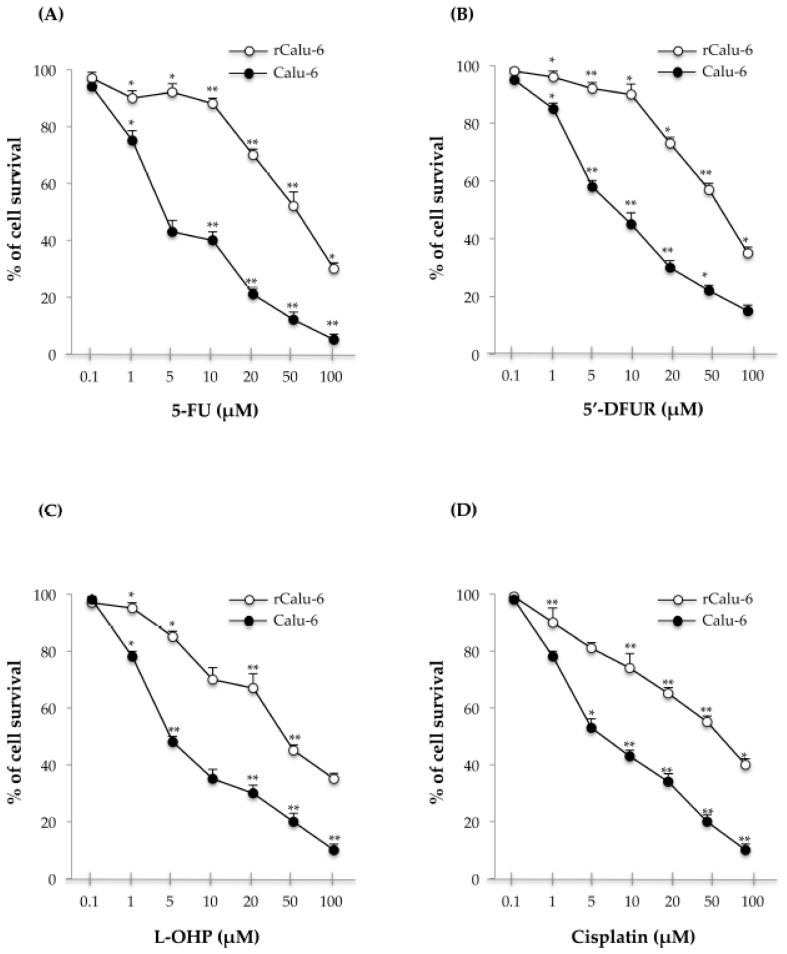
In vitro sensitivity of Calu-6 and rCalu-6 cells to 5-Fluorouracil (5-FU), 5′-Deoxy-5-fluorouridine (5′-DFUR), Oxaliplatin (L-OHP), and Cisplatin. Cells were cultured with increasing concentrations of 5-FU for 48 h and cell growth was assessed by sulforhodamine B (SRB) colorimetric assay. Cell growth was expressed as the percentage of control for each time point. ** *p* < 0.01, * *p* < 0.05 vs. untreated cells. The results illustrated in Figure 1, Figure 2, Figure 3, Figure 4 and Figure 5 are representative of three independently performed experiments; error bars represent the standard deviation.

To explore the mechanisms of MDR, we established a multidrug resistant Calu-6 subline (rCalu-6 cells) derived from the parental Calu-6 sensitive cells. To this aim, Calu-6 cells were continuously selected with an increasing concentration of 5-FU (from 2.5 to 25 µM) over a period of about 10 months in order to generate the corresponding age and passage-matched 5-FU resistant cell line designated rCalu-6. Maintenance of the resistant subline was continued at 20 µM. Figure 1 shows the cell survival curves of 5-FU sensitive and 5-FU resistant cells, and Calu-6 and rCalu-6 cells, respectively, after treatment with different concentrations of 5-FU for 48 h. Interestingly, the drug-adapted rCalu-6 cell line showed a significantly higher IC_50_ compared with parental Calu-6 cells (52.3 ± 0.02 vs. 4.5 ± 0.08, respectively). The degree of resistance to 5-FU was evaluated as the ratio of the IC_50_ values of 5-FU resistant Calu-6 cells to that of parental cells (Table 1). The results shown in Figure 1A and Table 1 indicate that the Calu-6 subline was successfully established as a 5-FU-resistant lung cancer cell line with a degree of resistance to 5-FU 11.7-fold higher than parental Calu-6 cells.

### 2.2. Cross-Resistance Profiles of rCalu-6 Cells

Since drug resistance involves the resistance to one drug accompanied by the resistance to several other anticancer drugs [29], we evaluated whether rCalu-6 cells acquired cross-resistance to other conventional anticancer drugs such as 5′-deoxy-5-fluorouridine (5′-DFUR) (Figure 1B), oxaliplatin (L-OHP, Figure 1C) and cisplatin (Figure 1D). Interestingly, cross-resistance to other classes of anti-cancer agents was observed, indicating potential multi-resistant phenotypes. The degree of resistance of rCalu-6 cells to 5′-DFUR, cisplatin, and L-OHP were 7, 6, and 10-fold higher than that of parental sensitive Calu-6 cells, respectively (Table 1). These results indicate that rCalu-6 cells show resistance to multiple drugs, representing an interesting tool to study MDR in lung cancer.

### 2.3. Expression Analysis of Genes Related to Drug Resistance in rCalu-6 Cells

**Figure 2:**
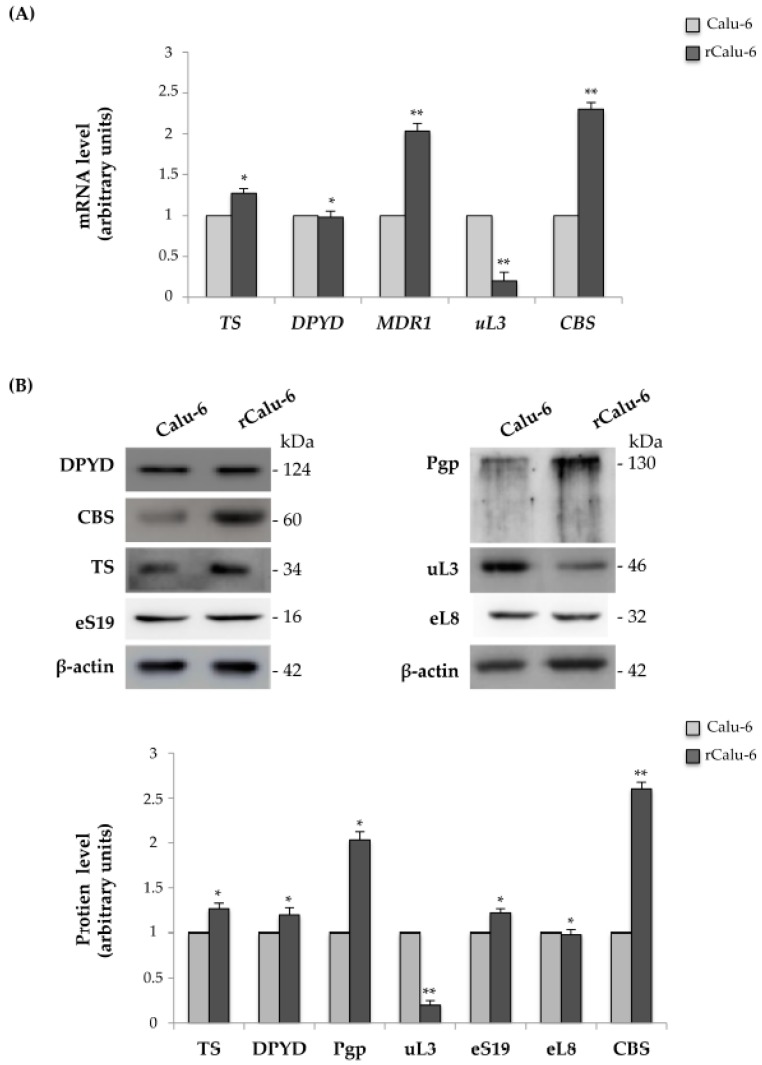
Analysis of mRNAs and proteins related to chemoresistance. (**A**) Total RNA from Calu-6 and rCalu-6 cells was subjected to Reverse Transcription quantitative Polymerase Chain Reaction (RT-qPCR) with primers specific for the indicated mRNAs. The quantification of signals is shown. ** *p* < 0.01, * *p* < 0.05 vs. mRNA levels in Calu 6 cells set at 1; (**B**) Protein extracts from Calu-6 and rCalu-6 cells were analyzed by Western blotting with antibodies against the indicated proteins. β-actin was used as the loading control. The quantification of signals is shown. ** *p* < 0.01, * *p* < 0.05 vs. protein levels in Calu 6 cells set at 1.

According to previous studies, 5-FU resistance is caused by an increase in the 5-FU-degrading enzyme dihydropyrimidine dehygrogenase (DPYD) and 5-FU-targeting enzyme thymidylate synthase (TS) [30]. On the other hand, ABC family proteins, such as Multi Drug Reactivity 1 (MDR1), are related to multiple drug resistance in cancer. We have previously demonstrated that uL3 is able to negatively control Pgp expression in colon cancer cells by regulation of *MDR1* mRNA stability [24]. Moreover, we also demonstrated that the loss of uL3 and the consequent upregulation of the cystathionine β synthase (CBS) enzyme correlated with ineffectiveness of 5-FU in lung and colon cancer cell lines [23]. Based on these data, we employed quantitative real-time PCR (qRT-PCR) to assess the expression profile of *TS*, *DPYD*, *MDR1*, *uL3*, and *CBS* in Calu-6 cells and rCalu-6 cells. As shown in Figure 2A, the resistant cell line showed an increase in *MDR1* and *CBS* mRNA amounts of about 2-fold compared to those observed in 5-FU sensitive Calu-6 cells. In contrast, for the condition of 5-FU resistance, the mRNA levels of *uL3* were strongly decreased in the resistant cell line compared to control cells.

The mRNA expression of *TS* and *DPYD* did not differ between drug resistant and sensitive cell lines. The expression of these genes at the protein level by Western blot analysis in Calu-6 and rCalu-6 cells was consistent with the mRNA analysis (Figure 2B). The expression levels of eS19 and eL8, two arbitrary proteins of large and small subunits, respectively, remained unchanged.

### 2.4. uL3 Mediates Anti-Oxidative Cell Response in rCalu-6 Cells

**Figure 3:**
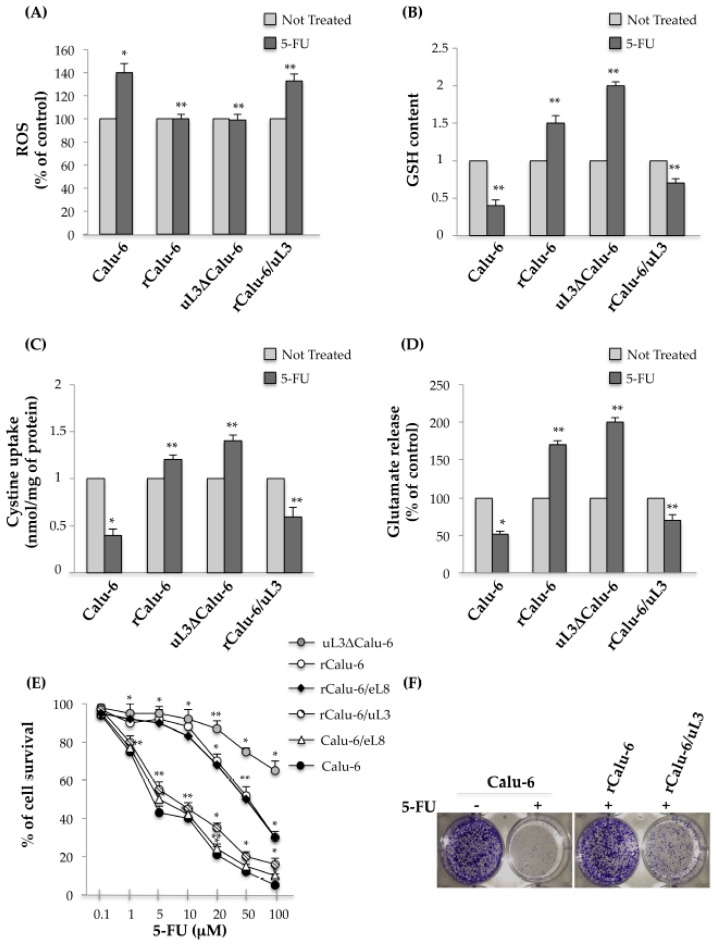
uL3 contributes to Reactive Oxygen Species (ROS) defense in rCalu-6 cells. (**A**) Histogram quantifying ROS production in indicated cell lines after treatment with 5-FU. ** *p* < 0.01, * *p* < 0.05 vs. respective untreated cells set at 1; (**B**) Glutathione (GSH) content in Calu-6 cells and derivative sublines after treatment with 5-FU. The quantification of signals is shown. ** *p* < 0.01, * *p* < 0.05 vs. respective untreated cells set at 1; (**C**) Changes in cystine uptake after incubation of indicated cells with 5-FU. ** *p* < 0.01, * *p* < 0.05 vs. respective untreated cells set at 1; (**D**) Evaluation of glutamate release in indicated cell lines after treatment with 5-FU. ** *p* < 0.01, * *p* < 0.05 vs. respective untreated cells set at 1; (**E**) uL3ΔCalu-6, rCalu-6, rCalu-6/uL3, rCalu-6/eL8, Calu-6/eL8 and Calu-6 cells were cultured with increasing concentrations of 5-FU for 48 h and cell growth was assessed by SRB colorimetric assay. Cell growth was expressed as the percentage of control for each time point. Ectopic expression of uL3 (rCalu-6/uL3 cells) abolished resistance to 5-FU. ** *p* < 0.01, * *p* < 0.05 vs. Calu 6 cells; (**F**) Representative image of the clonogenic analysis for cell proliferation of Calu-6, rCalu-6, and rCalu-6/uL3 after treatment with 10 μM 5-FU for 48 h.

It is known that the toxicity of antitumor drugs may largely depend on the redox status of the cells. The observed decreased expression of uL3 in rCalu-6 led us to hypothesize that the levels of uL3 would be functionally related to ROS production in these cells. To test this hypothesis, we first examined ROS production in Calu-6 cells and the resistant parental subline. To this aim, Calu-6 and rCalu-6 cells, were treated with 10 μM 5-FU for 48 h and then the ROS content was determined. As expected, we found that 5-FU treatment increased ROS production in 5-FU sensitive Calu-6 cells compared to the untreated cells, while in the resistant rCalu-6 cell line and uL3ΔCalu-6 cells, in which uL3 expression was stably switched off, 5-FU treatment failed to induce ROS production (Figure 3A). Next, we monitored the levels of intracellular GSH, that is known to play an important role in providing protection against oxidative damage in the same cells. As shown in Figure 3B, the GSH content in rCalu-6 and uL3ΔCalu-6 treated cells was improved compared with that found in the untreated cells. As expected, in treated Calu-6 cells the level of GSH was significantly lower than in the untreated cells. Next, since cystine is essential for the generation of GSH, we tested cystine uptake and the release of glutamate in the same cells. Figure 3C,D shows that cystine uptake and glutamate release were strongly inhibited in Calu-6 cells after drug treatment. On the contrary, the acquisition of drug resistance was associated to a significant increase of cystine uptake and glutamate release after 5-FU treatment. These data clearly suggest that oxidative stress target genes are involved in the molecular mechanism for acquiring MDR resistance in Calu-6 cells. Interestingly, we demonstrated that the observed alteration in the cell redox status of resistant cells was specifically mediated by uL3. In fact, the enforced expression of uL3 in rCalu-6/uL3 was associated to the loss of chemoresistance as observed by the inversion of the redox status in these cells (Figure 3A–D). Additionally, we performed cell proliferation assays to evaluate the cell response to 5-FU upon alteration of uL3 status in the cells. As shown in Figure 3E, the down-regulation of uL3 (rCalu-6 cells and uL3ΔCalu-6) was associated to a marked reduced cell response to 5-FU. The restoration of uL3 (rCalu-6/uL3) re-sensitized rCalu-6 cells to 5-FU causing a decrease of the percent of cell survival after 5-FU treatment. Interestingly, the over-expression of eL8 in rCalu-6 cells failed to overcome the chemoresistance and in Calu-6 cells did not affect the cell response to 5-FU, demonstrating the specificity of uL3 in these processes. The central role of uL3 in mediating the cell response to 5-FU was also confirmed by the results from the clonogenic survival assays (Figure 3E).

All together, these results demonstrated that uL3 is essential to mediate the cell response to 5-FU in Calu-6 cells by control of the cell redox status.

### 2.5. uL3 Reduces xCT and GST-α1 Expression Levels

**Figure 4:**
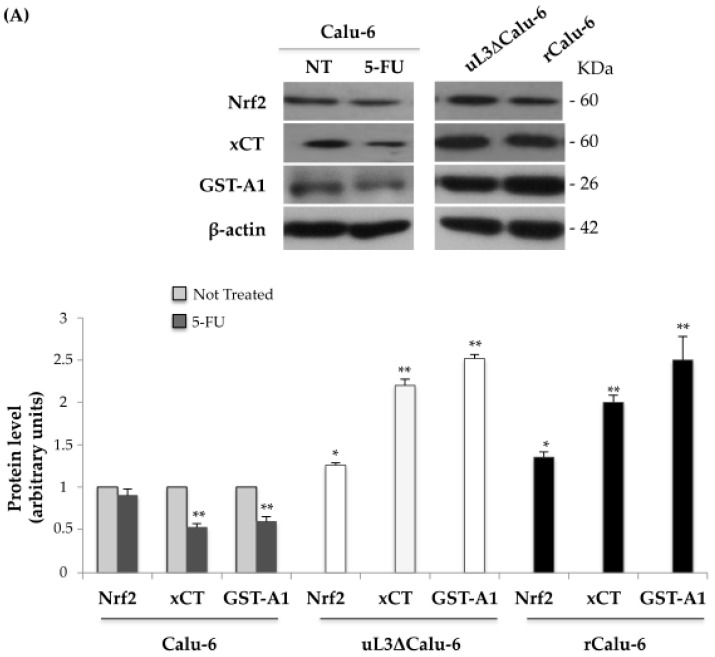

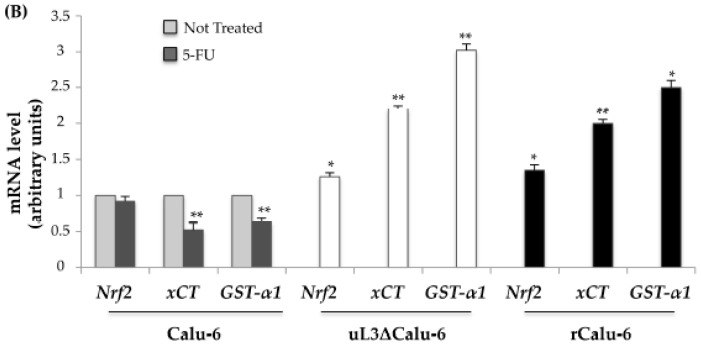
Influence of uL3 on Nrf2, xCT, and GST-A1 expression levels in Calu-6 cells and resistant sublines. (**A**) Protein extracts from Calu-6 cells treated or not with 10 μM 5-FU for 48 h, uL3ΔCalu-6 and rCalu-6 cells were analysed by Western blotting with the indicated antibodies. Anti-β-actin was used as the loading control. The quantification of signals is shown. ** *p* < 0.01, * *p* < 0.05 vs. protein levels in untreated Calu-6 cells set at 1; (**B**) Total RNA from Calu-6 treated or not with 10 μM 5-FU for 48 h, uL3ΔCalu-6 and rCalu-6 cells were subjected to qPCR with primers specific for the indicated genes. The quantification of signals is shown. ** *p* < 0.01,* *p* < 0.05 vs. mRNA levels in untreated Calu-6 cells set at 1.

To better characterize the molecular mechanism by which uL3 controls the alteration in cell-redox status in MDR resistance, we firstly assessed the intracellular levels of Nrf2 in drug sensitive and resistant Calu-6 cells, a crucial transcriptional regulator that activates various genes involved in oxidative stress, together with xCT and GST-α1, as representative Nrf2-regulated antioxidant defense/drug metabolism genes. To this aim, proteins extracted from Calu-6 treated or not treated with 10 μM 5-FU for 48 h, uL3ΔCalu-6, and rCalu-6 cells were analyzed by Western blotting with antibodies against Nrf2, xCT, and GST-A1. Figure 4A shows that in Calu-6 cells, Nrf2 levels are not significantly modified by 5-FU treatment while xCT and GST-A1 expression levels were markedly decreased. Interestingly, in uL3ΔCalu-6 and rCalu-6 cells, in which uL3 was respectively stably silenced and significantly down-regulated, we observed a marked increase of the xCT and GST-A1 levels. On the contrary, Nrf2 levels were not significantly altered in both cell lines and immunoprecipitation experiments demonstrated the absence of an interaction between Nrf2 and uL3 (Figure S1).

These results clearly demonstrate a central role of uL3 in the regulation of *xCT* and *GST-α1* expression in response to 5-FU treatment and in multidrug resistance.

Next, we analyzed the expression of *xCT* and *GST-α1* at the mRNA level. To this aim, the total RNA from Calu-6, treated or not treated with 10 μM 5-FU for 48 h, uL3ΔCalu-6, and rCalu-6 cells was isolated and analyzed by quantitative RT-PCR using primers specific for *Nrf2*, *xCT*, and *GST-α1* mRNAs (Figure 4B)*.* Consistent with the protein analysis, the treatment of Calu-6 cells with 5-FU resulted in a small reduction of the *Nrf2* mRNA level while *xCT* and *GST-α1* mRNA levels were significantly decreased. Interestingly, in uL3ΔCalu-6 and rCalu-6 cells the absence or the reduced expression of uL3, respectively, was associated to an increase in the *xCT* and *GST-α1* mRNA levels. The analysis of *xCT* and *GST-α1* mRNA levels in rCalu-6 over-expressing uL3 (rCalu-6/uL3) was consistent with these data (Figure S2A). In addition, we evaluated the influence of 5-FU on *xCT* and *GST-α1* mRNA levels in Calu-6, ΔuL3/Calu-6, and rCalu-6 cells. As expected, in the ΔuL3/Calu-6 and rCalu-6 cells, the levels of *xCT* and *GST-α1* were not decreased after 5-FU treatment, in contrast to Calu-6 cell line (Figure S2B).

Altogether these results indicate that uL3 was necessary for regulating *xCT* and *GST-α1* expression in response to 5-FU treatment and in drug resistance.

### 2.6. uL3 Is a Transcriptional Repressor of xCT and GST-α1

Recently, we have demonstrated that after drug treatment the ribosome-free uL3 behaves as a transcription factor [22], thus we became interested in determining if the uL3-mediated down-regulation of *xCT* and *GST-α1* expression occurred via the inhibition of gene transcription. To test the presence of uL3 on *xCT* and *GST-α1* gene promoters, Calu-6 cells untreated or treated with 10 μM 5-FU for 48 h, were collected and subjected to Chromatin immunoprecipitation experiments by using anti-uL3 antibodies and anti-IgG as negative control.

**Figure 5:**
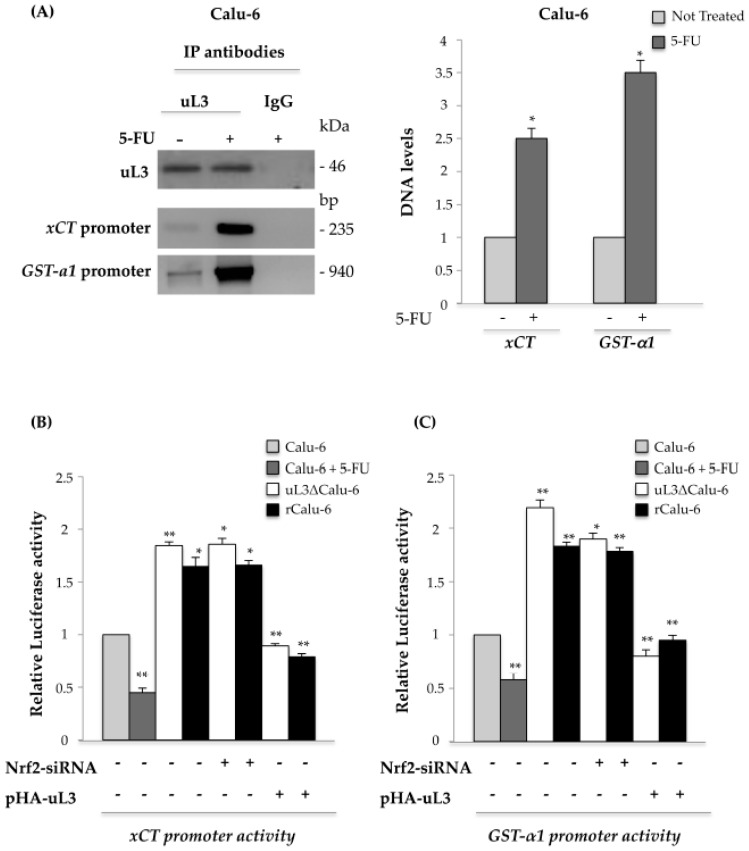
Analysis of the interaction between uL3 and *xCT* and *GST-α1* gene promoters. (**A**) Protein samples of DNA-uL3 or DNA-IgG immunocomplexes from Calu-6 cells untreated or treated with 10 μM 5-FU for 48 h were analysed by Western blotting with antibodies against uL3. Note the absence of signal in the DNA-IgG immunocomplex. The same DNA-immunoprecipitates were subjected to qPCR with primers specific for *xCT* or *GST-α1* gene promoters. The quantification of signals is shown. *p < 0.05 vs. DNA levels in untreated cells set at 1. Calu-6 cells treated or not treated with 10 μM 5-FU for 48 h, uL3ΔCalu-6 and rCalu-6 cells were transiently transfected with (**B**) *xCT* promoter luciferase reporter plasmid or (**C**) *GST-α1* promoter luciferase reporter plasmid in presence or absence of Nrf2 siRNA or pHA-uL3 plasmid. Analysis of the relative luciferase activity, normalized against Renilla Luciferase (pRL) activity, of the samples is shown. ** *p* < 0.01, * *p* < 0.05 vs. untreated Calu-6 cells set at 1.

The presence of uL3 in DNA-immunoprecipitated complexes was assayed by Western blotting (Figure 5A). qPCR assays on the samples were performed as previously reported [19]. Figure 5A shows that uL3 was able to bind to *xCT* and *GST-α1* promoters. Interestingly, after 5-FU treatment, this binding was significantly increased compared to that observed in the control, untreated Calu-6 cells.

Next, to test the role of uL3 on *xCT* and *GST-α1* promoter activities in the response to drug exposure and in the condition of acquired MDR resistance, we performed reporter luciferase assays. To this aim, Calu-6 cells untreated or treated with 10 μM 5-FU, rCalu-6, and uL3ΔCalu-6 cells were transiently transfected with the *xCT* (Figure 5B) or *GST-α1* (Figure 5C) promoter luciferase reporter constructs. Figure 5B,C shows that 5-FU treatment significantly reduced *xCT* and *GST-α1* promoter activity in Calu-6 cells. On the contrary, the transcriptional activity of these promoters was greatly increased in the condition of uL3 silencing (uL3ΔCalu-6 cells) and in rCalu-6 cells where uL3 expression was down-regulated. To demonstrate that the observed up-regulation of *xCT* and *GST*-α1 promoter activity in rCalu-6 and uL3ΔCalu-6 cells was specifically mediated by uL3 and independent from Nrf2, we performed analogous experiments in the conditions of Nrf2 silencing or uL3 overexpression. As shown in Figure 5B,C, Nrf2 depletion did not cause any alteration on *xCT* or *GST-α1* promoter activities. Ectopic expression of uL3 dramatically reduced *xCT* and *GST-α1* promoter trans-activation. These results strongly indicate that uL3 is an important player in the transcriptional regulation of *xCT* and *GST-α1* genes, acting as a negative regulator of their expression independently of Nrf2.

## 3. Discussion

In this study, we used human Calu-6 cells as a model system to establish a p53-mutated multidrug resistant lung cancer cell line. By the stepwise increase of the 5-FU concentration in the growth medium and selection of drug-resistance colonies for ten months, we successfully established a 5-FU resistant cell line, rCalu-6, with a resistance index of 11.7. In addition, rCalu-6 cells acquired cross-resistance to L-OHP and cisplatin. To the best of our knowledge, this is the first study on the production of a multidrug resistant Calu-6 cell line. This should be useful to study the mechanisms underlying drug resistance of *p53* mutated lung cancer.

The determinants of the resistance mechanisms to 5-FU include the enzymes involved in the 5-FU metabolism pathway [30]. To exert its cytotoxic effects, 5-FU is converted to its active metabolites by several enzymes and the levels of these enzymes have been associated with 5-FU sensitivity [6]. Previous results reported that the enhanced expression of dihydropyrimidine dehygrogenase (DPYD) and thymidylate synthase (TS), a 5-FU-degrading enzyme and a 5-FU-targeting enzyme, respectively, correlated with the development of 5-FU resistance [30]. However, we found that the expression of *DPYD* and *TS* was not enhanced in rCalu-6 cells. This suggests that the mechanisms of 5-FU resistance of rCalu-6 cells differ from those generally reported. Interestingly, the acquired drug resistance was associated to the modification of uL3 expression resulting in a decrease of its intracellular amount. We have also previously observed a reduction of uL3 levels in Cisplatin resistant A549 cells [25] that, unlike Calu-6 cells, are proficient for p53, suggesting that the loss of uL3 could be considered a general mechanism of multidrug resistance independent of p53 status. Furthermore, we demonstrated that the loss of uL3 was associated with the up-regulation of the *MDR1* mRNA level and Pgp transporter encoded by *MDR1* in colon cancer cells [24]. According to this, the multidrug resistance in rCalu-6 cells is associated to the high expression of the human *MDR1* gene and the Pgp protein levels.

It is known that the toxicity of antitumor drugs may largely depend on the intracellular level of reduced GSH that represents the first line antioxidative defense mechanism [31]. Increased resistance to cancer chemotherapeutic agents is often associated to elevated levels of GSH due to the protective conjugation and detoxification effects of GSH. To date, it has been demonstrated that the increase of GSH concentration or GST expression are linked to chemoresistance pathways in human breast cancer cells [32]. Pathways involved in GSH production and regeneration are controlled by Nrf2, a crucial regulator of the expression of different genes involved in oxidative stress response and drug resistance [33,34,35]. In order to evaluate the role of the GSH/GST system in rCalu-6 cells, we monitored the levels of intracellular GSH in these cells and in its parent cell line. rCalu-6 cells were shown to have higher GSH levels than the sensitive Calu-6 cells after 5-FU treatment. These effects are possibly mediated through up-regulated expression of xCT, causing an increase of the GSH level and inducing the antioxidative defense. xCT and Cystathionine gamma-lyase (CSE) are two important providers of intracellular cysteine, the precursor for the generation of GSH. xCT is the catalytic light chain of system xc^−^ cystine/glutamate antiporter, an Na^+^-independent heterodimeric amino-acid transport system that functions as the exchange system for the uptake of extracellular cystine in exchange for the release of intracellular glutamate, at a 1:1 ratio [36]. Once inside the cells, cystine is rapidly reduced to cysteine, the rate-limiting substrate for GSH synthesis involved in many physiological processes [37]. Our data showed that in rCalu-6 cells the cystine uptake and glutamate release were strongly increased, suggesting that oxidative stress target genes are involved in the molecular mechanism for acquiring multidrug resistance. We have previously demonstrated that uL3 potentiates the cytotoxicity of 5-FU [20,21,22,23] and we have also studied the effect of the 5-FU/uL3 combination by using specific delivery systems [38].

Of note, when uL3 was restored, rCalu-6 cells were resensitized to 5-FU, revealing that the restoration of uL3 could be a strategy for reversing 5-FU resistance and to resensitize 5-FU resistant tumors lacking p53 and uL3. Analysis of *xCT* and *GST-α1* mRNA levels reveales that the transcription of these genes is strongly up-regulated upon multidrug acquired resistance. In rCalu-6 cells, the down-regulation of uL3 is associated to an increase in *xCT* and *GST-α1* gene promoter activity. These two genes are known targets of Nrf2. The unchanged expression of *Nrf2* in rCalu-6 and the absence of an interaction between Nrf2 and uL3 (Figure S1) suggest an Nrf2-independent regulation of *xCT* and *GST-α1* gene expression in our experimental conditions. Accordingly, Nrf2 silencing in rCalu-6 cells did not cause any alteration on *xCT* and *GST-α1* promoter activity compared to cells expressing Nrf2. All together, these results indicate that uL3 controls the transcription of these genes independently of Nrf2. Although it is well established that cystine and glutamate are substrates of system xc^-^, it has been recently demonstrated that cystathionine is a novel substrate of the cystine/glutamate transporter [39]. Cystathionine-β-synthase (CBS), a key enzyme in the trans-sulfuration metabolic pathway involved in the modulation of H_2_S production [40,41], converts homocysteine to cystathionine, which is converted by CSE to cysteine required for synthesis of the antioxidant glutathione GSH [37]. Silencing CBS severely reduces cellular GSH levels [42]. We have previously demonstrated that uL3 is a negative regulator of CBS expression both at the transcriptional and post-translational levels [23]. rCalu-6 cells express low level of uL3 and high level of CBS. It is plausible that the observed increase of cellular GSH levels in rCalu-6 cells was also due to the loss of the CBS inhibitor uL3 with consequent activation of the trans-sulfuration pathway via CBS that lead to increased availability of the GSH precursor cysteine.

**Figure 6:**
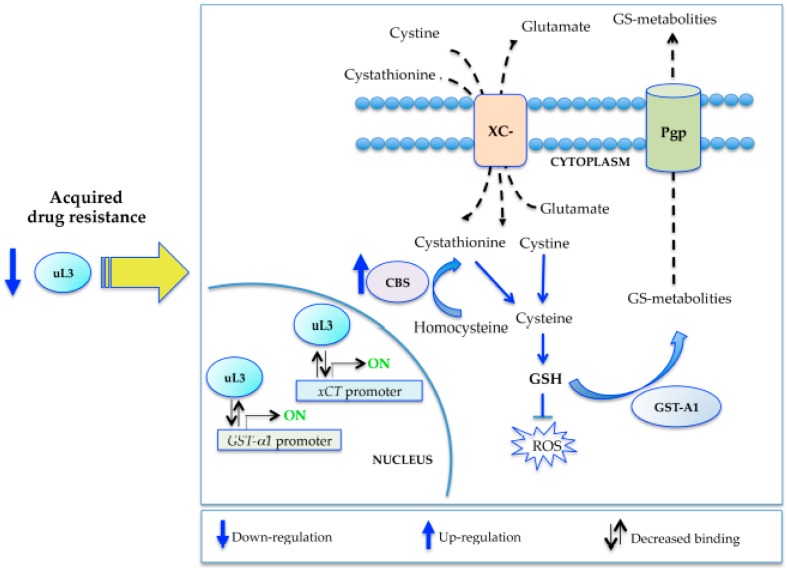
Proposed model of acquired multidrug resistance based on uL3 status.

All together, our results led us to propose a model by which upon acquired resistance, the activation of the defense response depends on the uL3 status (Figure 6). In particular, in the condition of multidrug resistance, the production of uL3 is down-regulated and the expression of stress-response genes is activated. Specifically, the binding of uL3 to *xCT* and *GST-α1* gene promoters decreases and, as a consequence, its inhibitory activity on these two promoters is suppressed. This represents an indirect way to potentiate the expression of antioxidant genes and activate the defense response. On the one hand, overexpression of the xCT light chain and the consequent increased activity of the antiporter system xc^-^ and, on the other hand, the activation of the trans-sulfuration pathway via CBS induced by lower levels of uL3 [22], lead to increased availability of Cysteine, a GSH precursor crucial for cell stress response. In addition, uL3 down-regulation positively associated to the up-regulation of Pgp-expression, contributing to the efflux mediated resistance (Figure 6).

## 4. Materials and Methods

### 4.1. Cell Cultures, Drug Treatment, and Production of Multidrug Resistant Calu-6 Cells

Human Calu-6 were cultured as previously described [43]. The uL3ΔCalu-6 cell line was obtained from Calu-6 cells by transfecting 2 μg of shRNA uL3 plasmid [21] by using Lipofectamin 2000 (Life Technologies, Carlsbad, CA, USA) according to the manufacturer’s instructions.

The multidrug resistant Calu-6 cell line (rCalu-6) was established from Calu-6 cells by continuous exposure to increasing concentrations of 5-FU (from 2.5 to 25 µM). rCalu-6 cells were then maintained in the presence of 20 µM 5-FU.

For drug treatments, rCalu-6 cells were grown in medium without 5-FU for 2 weeks before 5-FU addition.

### 4.2. Cell Viability Assay and Clonogenic Assay

The Sulforhodamine B (SRB) colorimetric assay for cell viability of Calu-6 and rCalu-6 cells and the clonogenic assay were performed as previously described [25].

### 4.3. Glutathione Measurement

Intracellular GSH concentration was measured using a commercially available kit (Chemicon, Temecula, CA, USA) according to the manufacturer’s protocol.

### 4.4. Glutamate Analysis

Glutamate in the cell medium was assayed with Amplex Red assay kits (catalog number: A-12,221, Invitrogen, Carlsbad, CA, USA) according to the manufacturer’s protocol. All treatments were performed in serum-free and L-glutamine-free MEM (Invitrogen).

### 4.5. Cystine Uptake

Cystine uptake was performed as previously described [44] by using 50 µm l-[35S]-Cystine (specific activity 40 mCi/mmol).

### 4.6. Reactive Oxygen Species (ROS) Assay

Cells were seeded at a density of 1 × 10^4^ in 96-well clear bottom black plates, allowed to equilibrate overnight, and were starved in serum free medium for 24 h followed by treatments. Intracelluar ROS levels were determined by using the Cell Meter™ Fluorimetric Intracellular Total ROS Activity Assay Kit*Red Fluorescence according to the manufacturer’s guideline (catalog number: 22901, AAT Bioquest^®^, Sunnyvale, CA, USA).

### 4.7. DNA Constructs, siRNA, and Transfection

DNA constructs containing *x-CT* and *GST-α1* promoters, the plasmid encoding Flag-Nrf2, and the plasmid encoding uL3-HA (pHA-uL3) and eL8 (pHA-eL8) were already available [21]. siRNA against Nrf2 was purchased by Santa Cruz Biotechnology (catalog number: sc-37030, Santa Cruz, CA, USA). Transfections were performed as previously described [45].

### 4.8. Dual Luciferase Assays

The luciferase assay was performed with a Dual-Luciferase reporter assay system (Promega, Madison, WI, USA) according to the manufacturer’s instructions. The firefly luciferase activity was normalized to *Renilla* internal control luminescence.

### 4.9. Chromatin Immunoprecipitation

The chromatin immunoprecipitation assay was performed as previously reported [19].

### 4.10. RNA Isolation, RT, and Quantitative Real-Time PCR

Total RNA from the cells was extracted as previously reported [46]. RT-qPCR was performed as previously reported [19]. The primers used for RT-qPCR were the following: *TS* (Forward: 5′-TTACCTGAATCACATCGAGC-3′ and Reverse: 5′-ATATCCTTCGAGCTCCTTTG-3′); *DPYD* (Forward: 5′-GTTCTGGCTACCAGGCTAT-3′ and Reverse: 5′-CATAAGGTGTTGTCCTGGAA-3′); *uL3* (Forward: 5′-CAAAGGCTACAAAGGGGT-3′ and Reverse: 5′-CTCAGTGCGGTGATGGTAG-3′); *MDR1* (Forward: 5′-ATGCCTTCATCGAGTCACTG-3′ and Reverse: 5′-TAACAAGGGCACGAGCTATG-3′); *Nrf2* (Forward: 5′-GAGAGCCCAGTCTTCATTGC-3′ and Reverse: 5′-TGCTCAATGTCCTGTTGCAT-3); *xCT* (Forward: 5′-AAACTTGCTAAGCTCTGTGTTGG-3′, Reverse: 5′-CACATCACATGTTTGTACACTCG-3′); *GST-α1* (Forward: 5′-CAGCAAGTGCCAATGGTTGA-3′ and Reverse: 5′-TATTTGCTGGCAATGTAGTTGAGAA-3′); *β-actin* (Forward: 5′ GGCGGCACCACCATGTACCCT-3′ and Reverse: 5′-AGGGGCCGGACTCGTCATACT-3′). The primers used for *CBS* were previously reported [22].

The association of uL3 with endogenous *x-CT* and *GST-*α*1* promoters was measured by qPCR on immunoprecipitated chromatin as previously described [47].

The PCR products were run on agarose gels and visualized by ethidium bromide staining.

### 4.11. Western Blot Analysis

Western blotting analysis was performed as previously reported [48]. The membranes were incubated with anti-uL3 and anti-eL8 (Primm, Milan, Italy), anti-xCT and anti-GST-A1, anti-TS, anti-DPYD and anti-Pgp (Abcam, Cambridge, MA, USA), anti-eS19 (Sigma-Aldrich, St Louis, MO, USA), anti-CBS, and anti-β-actin (Santa Cruz Biotechnology). Proteins were visualized with enhanced chemiluminescence [49]. Densitometric analysis was carried out using ImageJ software (Image 1.49v, available on: http://imagej.nih.gov/ij, National Institutes of Health, Bethesda, MD, USA).

### 4.12. Immunoprecipitation Assay

The immunoprecipitation assay was performed as previously described [50] by using anti-Flag (Santa Cruz Biotechnology). The beads were washed and boiled in the SDS sample buffer. The eluted proteins were loaded on 12% SDS-PAGE and analyzed by Western blotting.

### 4.13. Statistical Analysis

Statistical analysis was performed as previously reported [51]. Statistical comparisons were made by Student’s *t*-test and one-way ANOVA. *p* < 0.05 was considered significant, *p* < 0.01 was considered highly significant.

## 5. Conclusions

In conclusion, in this study we provide new insights concerning a rational applicability of the combined treatment based on 5-FU plus uL3 [24] as an individualized therapy for tumors lacking uL3, in an attempt to overcome drug resistance and yield better clinical outcomes.

## Figures and Tables

**Table 1:** Antiproliferative effect of anticancer drugs on Calu-6 and rCalu-6 cells. The tesistance index (RI) is reported as IC_50_ rCalu-6/IC_50_ Calu-6.

Drug	IC_50_ (μM)	r/Calu-6	*p*-Value (Calu-6 vs. rCalu-6)
	Calu-6		
5-FU	4.5 ± 0.08	52.3 ± 0.02 (RI = 11.7X)	<0.01
5′-DFUR	8.35 ± 0.06	56.7 ± 0.03 (RI = 7X)	<0.05
L-OHP	5 ± 0.07	50 ± 0.03 (RI = 10X)	<0.01
Cisplatin	10 ± 0.02	60 ± 0.05 (RI = 6X)	<0.01

## Supplementary Materials

Supplementary materials can be found at www.mdpi.com/1422-0067/18/3/547/s1.
