# Supplementary material for: Role of uL3 in Multidrug Resistance in p53-Mutated Lung Cancer Cells
Source: Int J Mol Sci. 2017 Mar 3;18(3):547. doi: 10.3390/ijms18030547 (PMC5372563; doi:10.3390/ijms18030547)
Supplement: Supplementary file 1 [file ijms-18-00547-s001.pdf]

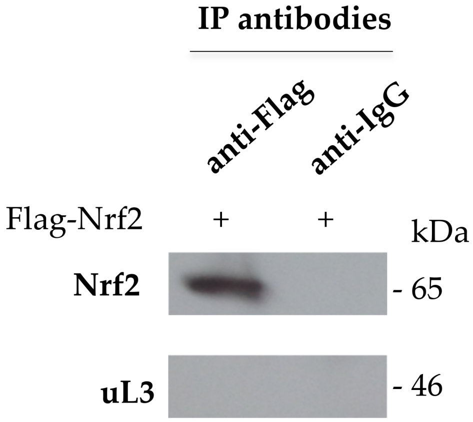

Figure S1

Figure S1. *In vivo* interaction between Nrf2 and uL3. Calu-6 cells were transiently transfected with a plasmid encoding Flag-Nrf2. Nrf2 was specifically immunoprecipitated with antibodies anti-Flag. The presence of Nrf2 and uL3 was tested with antibodies against the endogenous Nrf2 and uL3. No interaction between Nrf2 and uL3 was found.

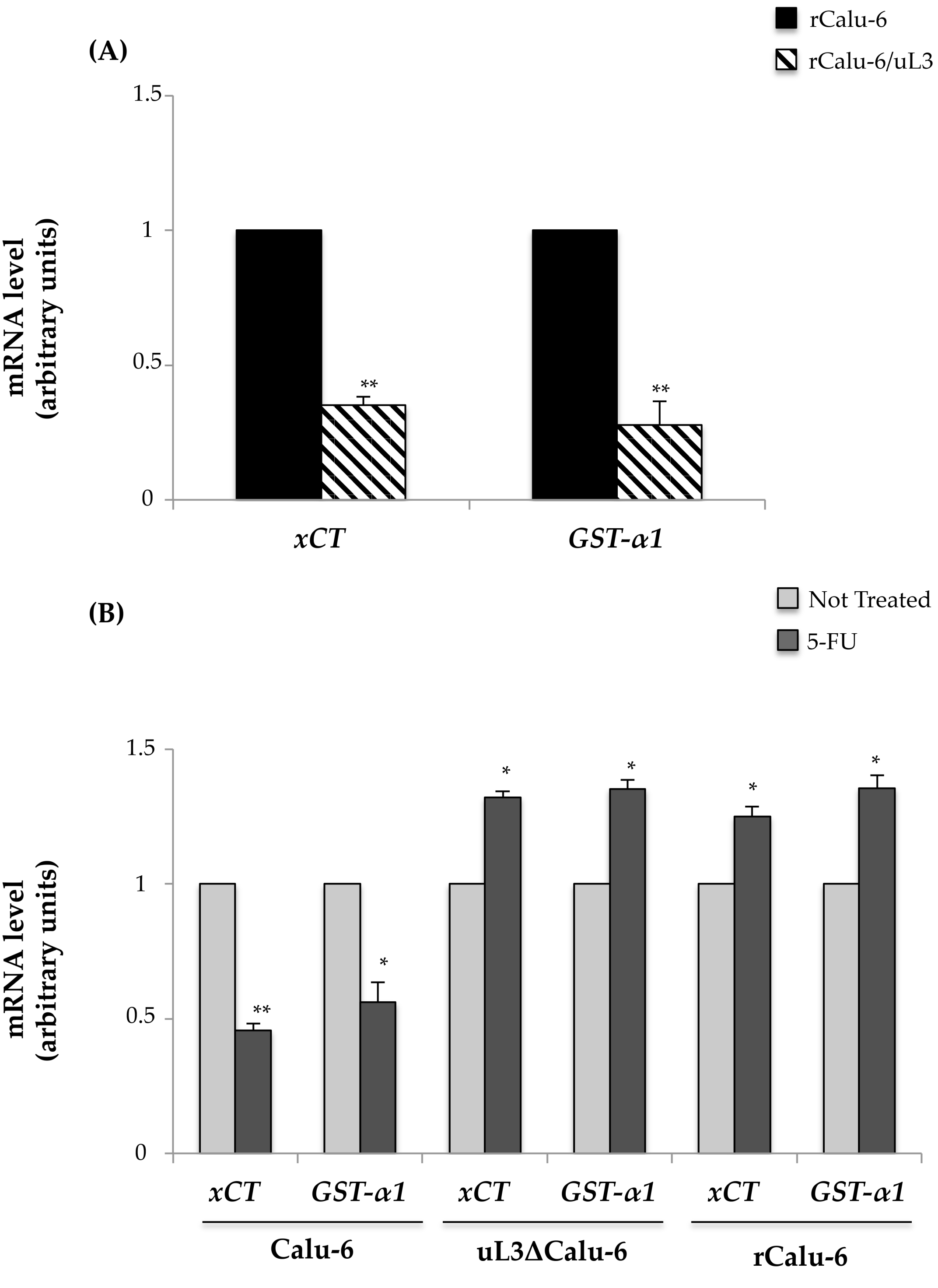

Figure S2. (**A**) Total RNA from rCalu-6 and rCalu-6/uL3 cells was subjected to RT-qPCR with primers specific for indicated mRNAs. Quantification of signals is shown. ** $p < 0.01$, * $p < 0.05$ vs. mRNA levels in rCalu-6 cells set at 1. (**B**) Calu-6, uL3ΔCalu-6 and rCalu-6 cells were treated with 10 μM 5-FU for 48 h and then extracted total RNA was subjected to RT-qPCR with primers specific for indicated mRNAs. Quantification of signals is shown. ** $p < 0.01$, * $p < 0.05$ vs. mRNA levels in untreated cells set at 1.

Figure S2
